# Implantable Cardioverter Defibrillator Generator Replacement and Breast Implant Revision—A Combined Case

**DOI:** 10.1002/ccr3.70132

**Published:** 2025-01-31

**Authors:** Vasileios Lamprou, John Murphy, Andrea Ardizzone, Niall G. Campbell

**Affiliations:** ^1^ Manchester Heart Institute, Wythenshawe Hospital Manchester University NHS Foundation Trust Manchester UK; ^2^ The Nightingale Centre and Genesis Prevention Centre, Wythenshawe Hospital Manchester University NHS Foundation Trust Manchester UK; ^3^ East Lancashire Hospitals NHS Foundation Trust Blackburn UK; ^4^ Faculty of Biology, Medicine and Health The University of Manchester Manchester UK

**Keywords:** breast implant, cardiac implantable electronic device, generator replacement, implantable cardioverter defibrillator

## Abstract

Physicians are increasingly likely to encounter patients with both cardiac implantable electronic devices (CIED) and breast implants in situ. Our case indicates the importance of appropriate planning and multidisciplinary input for CIED procedures in patients with breast implants or vice versa. When planning the procedure, the aesthetic outcome needs to be considered.

**Abbreviations**
CIED: cardiac implantable electronic devices
ICD: implantable cardioverter defibrillator

## 1 Introduction

Breast augmentation is the most common aesthetic procedure in the United Kingdom [1]. With more than half a million of the population in the United Kingdom having a CIED [2], an increasing number of female patients are expected to require both a CIED and breast augmentation revision or reconstruction procedure after mastectomy. These procedures involve the same anatomical regions and clinicians with specialist expertise in both CIEDs and breast surgery need to be aware of the potential challenges of managing such patients.

We report a case of a female with bilateral breast implant reconstruction and an implantable cardioverter defibrillator (ICD) requiring generator replacement. A joint case between a breast surgeon and CIED specialist cardiologist facilitated an optimal aesthetic and functional result.

## 2 Case History

A female with small stature in her late 50s had bilateral mastectomies as a prophylactic operation in 2011 and immediate reconstruction with breast implants which were placed in a submuscular plane.

A few years later, she experienced an anterior myocardial infarction and was treated with primary percutaneous intervention to the left anterior descending artery. This event resulted in severe left ventricular dysfunction that persisted despite optimal heart failure treatment and in 2017, a dual chamber ICD was inserted on primary prevention grounds.

For this procedure, a DDDR Autogen MINI ICD (Boston Scientific, USA) was implanted in a subcutaneous pocket in a standard fashion. A Mini ICD generator was selected instead of a standard size ICD given her small stature, minimal excess tissue, and the presence of breast implants.

Early post procedure, she experienced discomfort, and in 2019, after she lost weight, the patient reported that she had concerns about the aesthetic result. The lateral edge of the defibrillator was prominent and uncomfortable with movement and the lower margin of the generator was sited anterior to her left breast implant reconstruction.

A cardiologist and a breast surgeon consultant reviewed her jointly in clinic. After discussion with the patient, it was decided that she would be best served by a combined procedure to remove the left‐sided breast implant from its current subpectoral plane and place it in front of the pectoralis muscle (so‐called prepectoral technique), beneath a further layer of acellular dermal matrix; which had the effect of allowing the defibrillator to be sited deep to the breast implant on top of the pectoralis muscle. The procedure alleviated her discomfort from the CIED and the patient reported she was pleased with the aesthetic result.

## 3 Methods (Investigation and Treatments)

She was followed up in the cardiac devices clinic and early 2024 the CIED reached recommended replacement time when she was listed for generator change. She was keen to continue to have an ICD in situ despite not having any appropriate treatments from the device. Once again, a joint case between a breast surgeon and cardiologist was arranged to allow generator change minimizing the risk of implant complications. Due to the marginal increase in size between an Autogen MINI generator (Boston Scientific, USA; 26.5 cc, 9.9 mm) and a Resonate extended longevity ICD (Boston Scientific, USA; 29.5 cc, 9.9 mm), the decision was made to implant the extended longevity ICD to allow longer interval between the need for another generator change. This would therefore require a smaller breast prosthesis to ensure breast size symmetry.

**FIGURE 1:**
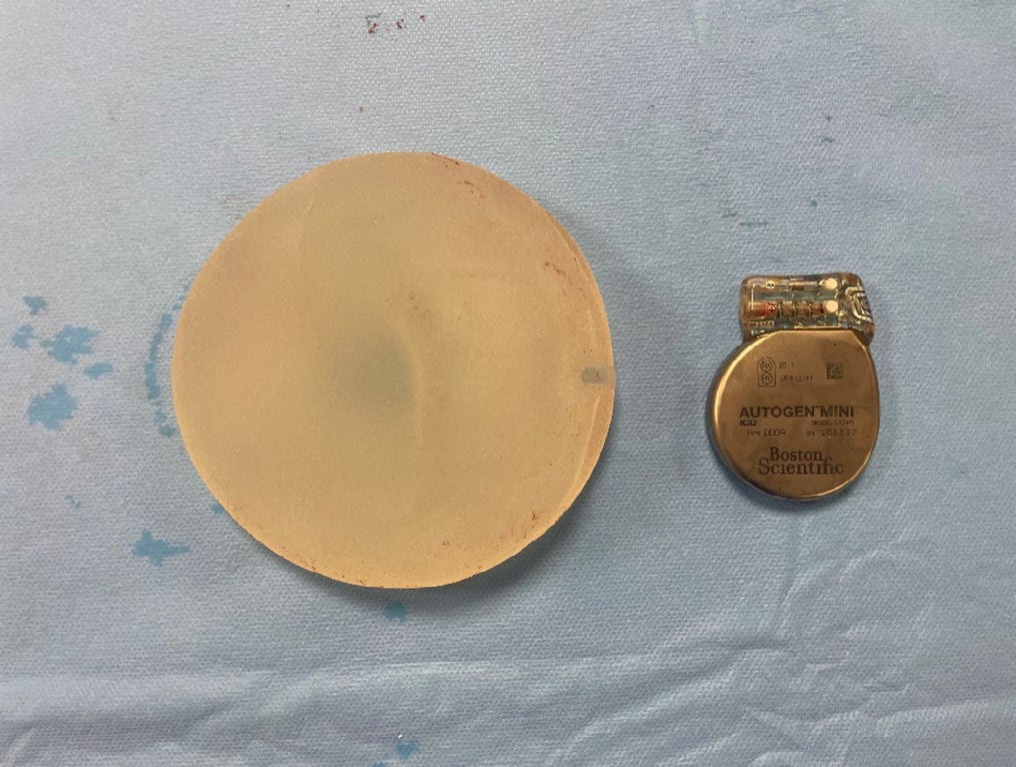
Explanted breast implant and Boston MINI ICD in comparison.

**FIGURE 2:**
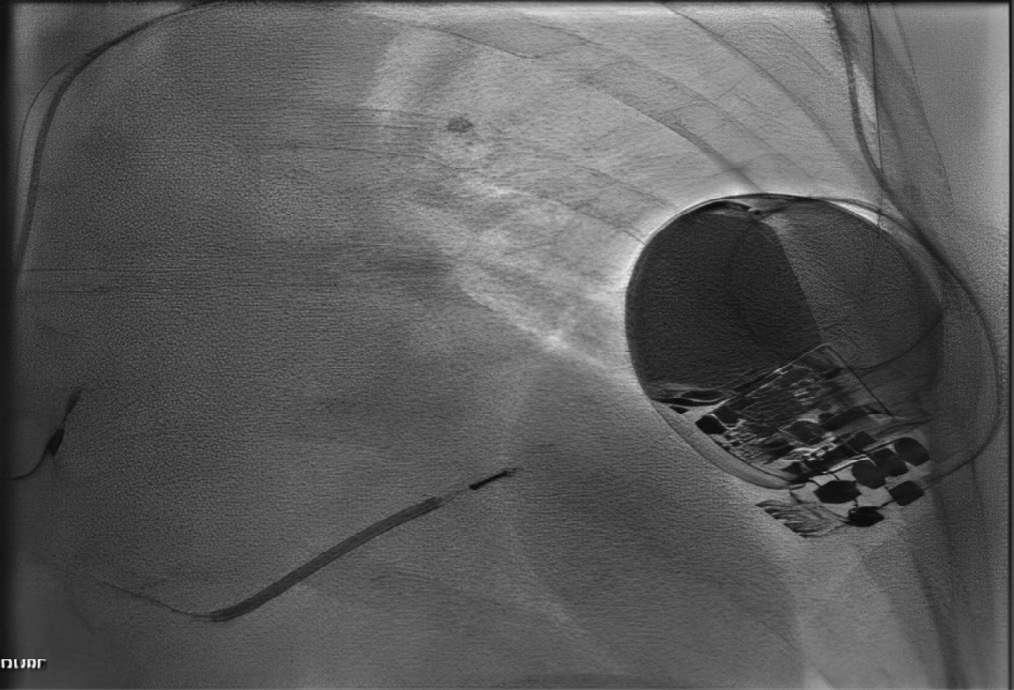
The new cardiac implantable electronic device generator lies underneath the breast implant.

The patient consented for the procedure, and she was admitted electively to the catheter lab. She was prepped and draped allowing access above and below the breast implant. The procedure was performed with local anesthetic and conscious sedation. An inframammary crease elliptical incision was made excising her existing scar caudal to her breast prosthesis. The prosthesis was mobilized, the old generator was removed, and the new generator was connected to her old leads (Figure 1). The CIED was then placed in an antimicrobial pouch (Tyrx, Medtronic, USA) to reduce the risk of infection and the generator was sutured to the underlying fascia at the cranial aspect of her prosthesis aiming to obtain a good ICD shock vector because her shock lead tip was previously sited close to her generator (Figure 2). A new smaller breast prosthesis was implanted above the ICD generator. The wound was closed in layers with Monocryl 3–0 and Glue. The ICD was reactivated, and all checks were satisfactory.

## 4 Conclusions (Outcome and Follow‐Up)

The wound had healed well when she was seen in the 6‐week cardiac device check in the clinic. She was also reviewed 6 months post‐procedure in clinic and reported that she was pleased with the aesthetic results.

## 5 Discussion

**FIGURE 3:**
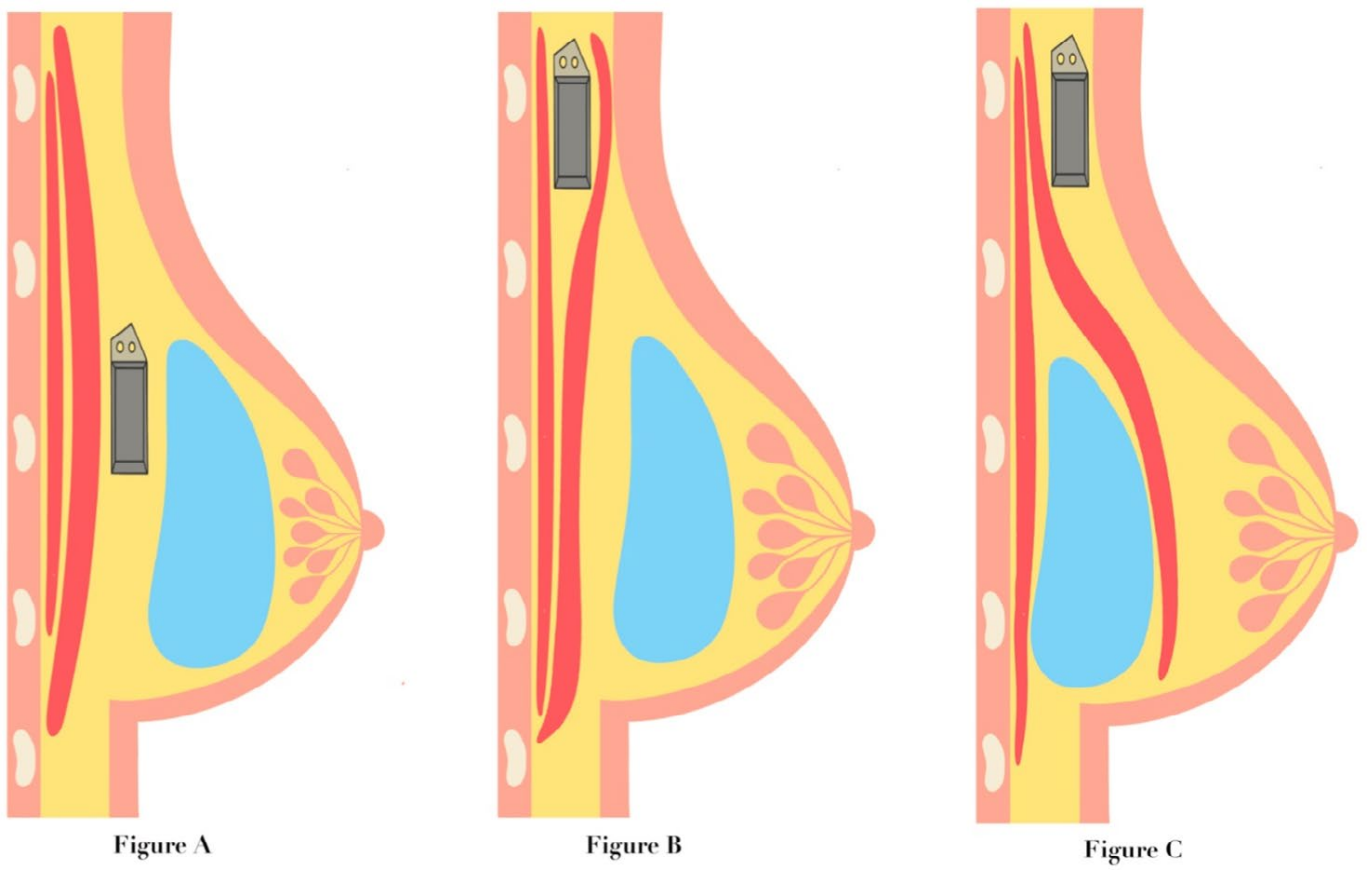
Fascial plane alternatives for combination of a cardiac implantable electronic device (CIED) generator and a breast implant. (A) CIED generator is sited below the breast implant. Both the CIED generator and the breast implant lie on the same subglandular plane (as performed in our case). The CIED generator was fixed with a suture to the muscle to avoid excess movements. (B) The CIED generator is implanted in a submuscular pocket and the breast implant is sited in a subglandular plane. (C) The CIED generator is sited in a subcutaneous pocket and the breast implant is positioned in a submuscular plane. This approach led to discomfort and poor aesthetic outcome in our case and is not recommended.

Both CIED and breast implants can be placed above and below the pectoralis major muscle and require special consideration when a fascial plane is shared. The different planes that can be used for both implants are shown in Figure 3. Although successful cases of combined CIED and breast implantation performed jointly by surgeons and cardiologists have been reported [3, 4], to the best of our knowledge, this is the first report of a multidisciplinary approach where the different size of the new ICD generator necessitated a simultaneous revision of the breast implant to ensure a good cosmetic result via a single submammary incision and allow the two implants to lie safely on the same plane (Figure 3A).

CIED implantation is usually performed through a horizontal incision inferior to the clavicle or an oblique incision along the deltopectoral groove. Inframammary and axillary approaches are used rarely and mainly for cosmetic reasons [5, 6]. In most patients, operators opt to implant the CIED generator in a subfascial prepectoral pocket [7]. For patients with minimal adipose tissue at risk of CIED erosion or with aesthetic concerns, submuscular pocket sited, below the pectoralis muscle are preferred [8]. The aesthetic outcome needs to be considered, especially in young or female patients who have a greater level of body image concerns post implant [9].

Cosmetic breast implants can be placed below the pectoris muscle (submuscular plane) or above the muscle (known as the subglandular plane) which may depend on patients’ preference and characteristics [10]. Submuscular implantation is associated with a reduced risk of infection; however, an increased risk of breast implant rupture has been described with older submuscular silicone implants [11]. Subglandular implants are associated with a more rapid recovery, although there is a higher risk of capsular contractures, hematomas, and seromas [10, 12]. The incision could be inframammary, transaxillary, or peri‐aleora. Based on patient characteristics and preferences, it may be most appropriate for them to receive a breast implant in a subglandular plane and a CIED implant in a submuscular plane (Figure 3B). However, a combined subpectoral breast augmentation and ICD implantation has also been reported resulting to excellent aesthetic outcome [4].

Complications in patients with CIED implants and breast implants have previously been recorded. A silicone breast implant rupture has been reported after the insertion of a defibrillator device when both devices were implanted on the same subglandular plane [13]. A case of subpectoral migration of the CIED generator into the breast implant pocket has also been reported requiring the placement of an acellular biologic matrix to support the CIED generator [14]. In both cases, the initial CIED procedure resulting in the complication was not planned or performed with a breast surgeon.

Our case demonstrates that frequently, it is inadvisable to place a CIED implant in a subcutaneous plane when the breast implant is already sited in a submuscular plane, as occurred initially, especially in a patient with no adequate subcutaneous tissue to cover the CIED (Figure 3C). We suggest that a detailed pre procedure consultation with the patient and a multidisciplinary approach with CIED specialists and breast/plastic surgeons to allow appropriate planning is required to ensure optimal management of patients with existing breast implants requiring a CIED intervention or vice versa, to maximize the likelihood of satisfactory aesthetic and safe outcomes [15].

Availability of both specialities will not be present in all centers. Regional centers of excellence with the relevant expertise need to be developed, along with appropriate referral systems, local education and working relationships. Renumeration and funding issues will also need to be considered, depending on local healthcare systems.

## Author Contributions

**Vasileios Lamprou:** conceptualization, writing – original draft. **John Murphy:** writing – review and editing. **Andrea Ardizzone:** writing – review and editing. **Niall G. Campbell:** writing – review and editing.

## Consent

Written informed consent was obtained from patient to publish this report in accordance with the journal's patient consent policy.

## Conflicts of Interest

The authors declare no conflicts of interest.

## Data Availability

The authors have nothing to report.
